# Novel DNA Barcoding and Multiplex PCR Strategy for the Molecular Identification and Mycotoxin Gene Detection of *Fusarium* spp. in Maize from Bulgaria

**DOI:** 10.3390/mps8040078

**Published:** 2025-07-09

**Authors:** Daniela Stoeva, Deyana Gencheva, Georgi Radoslavov, Peter Hristov, Rozalina Yordanova, Georgi Beev

**Affiliations:** 1 Department of Biological Sciences, Faculty of Agriculture, Trakia University, Students Campus, 6000 Stara Zagora, Bulgaria; daniela.stoeva@trakia-uni.bg; 2 Department of Fundamental Sciences in Animal Husbandry, Faculty of Agriculture, Trakia University, Students Campus, 6000 Stara Zagora, Bulgaria; deyana.gencheva@trakia-uni.bg; 3 Department of Animal Diversity and Resources, Institute of Biodiversity and Ecosystem Research, Bulgarian Academy of Sciences, 1113 Sofia, Bulgaria; gradoslavov@gmail.com (G.R.); peter_hristoff@abv.bg (P.H.); 4 Medical College, Trakia University, 9 Armeiska Street, 6000 Stara Zagora, Bulgaria; rozalina.yordanova@trakia-uni.bg

**Keywords:** *Fusarium* spp., maize, DNA barcoding, multiplex PCR, mycotoxigenic potential

## Abstract

*Fusarium* spp. represent a critical threat to maize production and food safety due to their mycotoxin production. This study introduces a refined molecular identification protocol integrating four genomic regions—ITS1, IGS, *TEF-1α*, and *β-TUB*—for robust species differentiation of *Fusarium* spp. isolates from post-harvest maize in Bulgaria. The protocol enhances species resolution, especially for closely related taxa within the *Fusarium fujikuroi* species complex (FFSC). A newly optimized multiplex PCR strategy was developed using three primer sets, each designed to co-amplify a specific pair of toxigenic genes: *fum6/fum8*, *tri5/tri6*, and *tri5/zea2*. Although all five genes were analyzed, they were detected through separate two-target reactions, not in a single multiplex tube. Among 17 identified isolates, *F. proliferatum* (52.9%) dominated, followed by *F. verticillioides*, *F. oxysporum*, *F. fujikuroi*, and *F. subglutinans*. All isolates harbored at least one toxin biosynthesis gene, with 18% co-harboring genes for both fumonisins and zearalenone. This dual-protocol approach enhances diagnostic precision and supports targeted mycotoxin risk management strategies.

## 1. Introduction

Maize (*Zea mays* L.) occupies a pivotal position in global agronomy, functioning as an essential staple in human nutrition, a principal constituent in animal feed, and a significant precursor for industrial applications [1,2,3,4]. Despite its status as a staple crop on a global scale, maize exhibits considerable vulnerability to fungal afflictions throughout both the cultivation and subsequent postharvest periods. Among the most troubling of these fungi are *Fusarium* species, which inflict damage on the grain while also producing harmful mycotoxins, including fumonisins (FUMs), zearalenone (ZEA), and trichothecenes (TRIs) [5,6]. These harmful toxins are increasingly recognized for their association with grave health complications in humans and animals, which may include liver damage, lowered immunity, neurological issues, and cancer [7,8,9].

Accurately discerning among *Fusarium* species is rendered complex by their morphological resemblances, the existence of cryptic species, and the heterogeneity of closely allied genera—exceeding twenty within the extensive fusarioid complex [10,11]. To surmount these challenges, molecular methodologies have increasingly been recognized as instrumental for species-level classification and elucidating evolutionary interrelations. In this inquiry, we employed a multilocus DNA barcoding methodology that, during the inaugural time, amalgamated four genetic loci—ITS1, IGS, *TEF-1α*, and *β-TUB*—to enhance the precision of *Fusarium* species classification. These genetic markers have demonstrated efficacy in distinguishing species within prominent *Fusarium* lineages, including the *F. fujikuroi* (FFSC), *F. oxysporum* (FOSC), and *F. sambucinum* (FSAMSC) species complexes [12,13,14].

Concurrently with species classification, evaluating the mycotoxigenic potential of *Fusarium* isolates is imperative for food safety. For this objective, we established and validated a multiplex polymerase chain reaction (PCR) assay targeting five genes associated with toxin biosynthesis: *fum6*, *fum8*, *tri5*, *tri6*, and *zea2*. This strategy facilitated simultaneous screening for genes implicated in the biosynthesis of principal mycotoxins, thereby bolstering prompt detection and remediation of potential contamination [15,16,17].

To date, this represents the inaugural study in Bulgaria to employ a synthesis of multilocus genetic identification and multiplex PCR analysis to characterize *Fusarium* spp. populations in maize grain. The objective of this research was to identify distinct *Fusarium* spp. isolates utilizing four genetic markers, investigate their phylogenetic interrelations, and evaluate their potential to synthesize mycotoxins through targeted molecular techniques.

## 2. Experimental Design

### 2.1. Sample Collection

**Figure 1:**
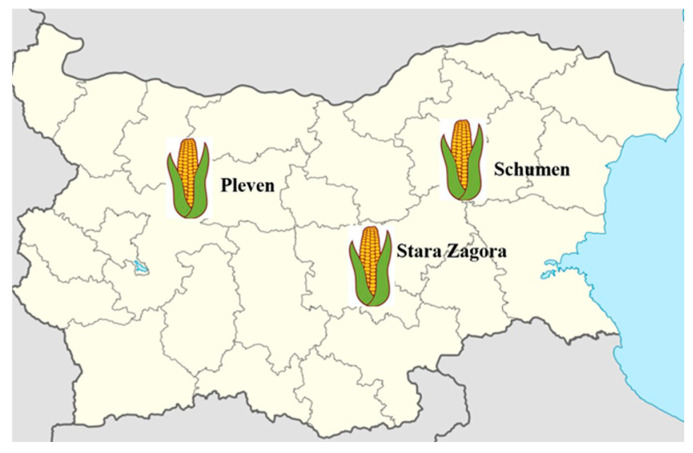
Geographical locations indicating the sampling sites of *Fusarium* spp. isolates.

A total of 50 maize kernel samples were collected during the period September–November 2023 from 3 main commercial maize-growing regions in Bulgaria (Figure 1)—Pleven (26 samples), Shumen (16 samples), and Stara Zagora (8 samples), from storage facilities and according to ISO 24333:2009 [18]. Briefly, 1 kg spot samples of maize with no visible disease symptoms were taken with a bulk profile sampler from 10 points throughout the bulk lot (lots ranged from 5 to 100 tons). Multiple samples from appropriate sites were aseptically poured into a sterile container and manually mixed to obtain a homogeneous composite sample. Following their complete mixing, a bulk of samples weighing 10 kg was formed and manually reduced to samples of 0.5 kg, which were then labeled. Sampling at storage facilities was chosen to focus on *Fusarium* spp. that persist post-harvest and present a direct risk to food and feed safety. This strategy reflected the study’s aim of assessing contamination under realistic storage conditions. While field sampling may reveal broader pre-harvest fungal diversity, post-harvest sampling provides relevant insights into the fungal populations that are most likely to enter the food chain.

### 2.2. Culture Media and Morphological Characterization of Fusarium spp. Isolates

One hundred maize kernels from each sample underwent random selection and surface sterilization in 70% ethanol for five minutes, followed by three rinses with distilled water to eliminate residual microflora. The kernels were dried with sterile filter paper and placed in 4 petri dishes (d = 15 cm) (25 grains in each) on PDA medium (HiMedia©, Maharashtra, India). Inoculated plates were incubated at 22–25 °C for 7 days [19], followed by calculation of the number of growing colonies belonging to *Fusarium* spp. The identification of *Fusarium* spp. was done using keys by Burgess et al. [20], Gerlach and Nirenberg [21], and Leslie and Summerall [22]. All suspected *Fusarium* spp. colonies were subcultured using the single spore technique [23]. A spore suspension was prepared in a 10 mL sterile water sample so as to contain 1 to 10 spores. Water agar (WA) plates were inoculated with 0.1 mL of the spore suspension and incubated at 25 °C for 18–20 h. To facilitate molecular identification, germinated single spores were subsequently transferred to synthetic nutrient-poor agar (SNA) and incubated for seven days at 25 °C, following the procedure outlined by Gerlach and Nirenberg [21]. Each isolate was then examined microscopically to assess the spore morphologies characteristic of *Fusarium* spp.

## 3. Procedure

### 3.1. DNA Extraction, PCR Amplification and Sequencing

A 5-day-old mycelium (1.5 ± 2 cm in diameter) of *Fusarium* spp. strains on SNA plates was used for genomic DNA isolation. Initially, to lysate the fungi’s polysaccharide cell wall, the mycelium was frozen for 24 h at −20 °C and then pulverized in conical Eppendorf tubes with quartz sand. DNA extraction was performed by using a Tissue DNA Preparation–Column Kit (Jena Bioscience GmbH, Jena, Germany) according to the manufacturer’s instructions. The concentration of the extracted genomic DNA of each sample was measured using a NanoView Plus spectrophotometer (GE HealthCare Technologies, Inc. Chicago, IL, USA) at a 260–280 nm wavelength. The DNA concentration of all samples was adjusted to 10 ng/µL, in a working volume of 70 µL, and the genomic DNA-extracted samples were stored at −18 °C until further analysis. The primers’ optimal annealing temperature was previously determined by multiple PCR amplification of the same sample in the temperature range 51.1–61.2 °C, and it was 52.5 °C for the ITS primers, 54.0 °C for the *TEF1-α* primers, 51.1 °C for the *β-TUB* primers, and 56.6 °C for the IGS primers (Table 1). Amplification was performed in a thermal cycler (QB-96 Thermal Cycler, Quanta Biotech Ltd., Surrey, UK), under the following conditions: initial denaturation at 95.0 °C for 5 min, followed by 30 cycles (denaturation at 95.0 °C for 0.30 min, primers annealing at specific temperature of target genes for 0.45 min, extension at 72.0 °C for 1 min), and final extension at 72.0 °C for 9 min.

### 3.2. Molecular Identification of Mycotoxigenic Potential of Identified Fusarium spp.

To evaluate the toxigenic potential of *Fusarium* spp. isolates, we developed and optimized a multiplex PCR approach targeting key genes from the biosynthetic gene clusters (BGCs) responsible for mycotoxin production. The assay consisted of three separate multiplex PCR reactions, each designed to co-amplify a specific pair of genes: *fum6/fum8*, *tri5/tri6*, or *tri5/zea2*. These gene pairs were selected based on primer compatibility in terms of annealing temperature and amplification efficiency. Each reaction was independently optimized to minimize primer–dimer formation and ensure robust co-amplification. This strategy enabled efficient and reliable screening of five mycotoxin biosynthesis genes across multiple isolates using three distinct two-gene multiplex assays.

For each PCR tube, 10 µL of genomic DNA was combined with an equal volume of Red Taq 2× Master Mix (VWR International BV, Leuven, Belgium). Magnesium chloride (VWR International BV, Leuven, Belgium) was subsequently incorporated at a concentration of 1.5 mM. Additionally, 0.8 µL of both forward and reverse primers were added, along with 1.8 µL of nuclease-free water, achieving a total volume of 25 µL. To establish the optimal annealing temperatures for the primers, we conducted multiple test reactions across a gradient of 51.1 to 61.2 degrees Celsius. The comprehensive list of primers and their corresponding optimal temperatures is detailed in Table 2. The degenerate primers used in this study were originally developed by Dawidziuk et al. [17] based on conserved motifs across multiple *Fusarium* species. To ensure optimal performance, all primers were further evaluated in silico for secondary structure formation (homodimer/heterodimer potential) using both in-house Python scripts and the IDT OligoAnalyzer Tool 1.0, employing nearest neighbor entropy/enthalpy models. According to Dawidziuk et al. [17] these primers demonstrated high sensitivity and specificity across multiple mycotoxin groups: trichothecenes (100% sensitivity, 95% specificity), zearalenone (100%/100%), and fumonisins (94%/88%), validating their robustness for detecting mycotoxigenic potential in mixed *Fusarium* populations.

While this protocol enabled preliminary screening of toxigenic potential, no expression or quantitative analysis (e.g., qPCR or ELISA) was performed in this study. As such, the assay assessed the *presence* of biosynthetic genes, not actual toxin production.

The PCR mixtures contained the same components as those for molecular detection of *Fusarium* spp. PCR amplifications were performed under the following conditions: initial denaturation for 30 s at 95.0 °C, 30 cycles of 30 s at 95.0 °C, primer annealing of 45 s at 51.4 °C (*fum6*/*fum8*), at 54 °C (*tri5/tri6*), or at 51. 9 °C (*tri5/zea2*), elongation of 1 min at 72.0 °C, and final extension of 9 min at 72.0 °C.

The obtained PCR products were separated on a 1% agarose gel electrophoresis (TopVision agarose, Fermentas, San Francisco, CA, USA) stained with 10,000× GelRed™ (Cat. No. 41003, Biotium Inc. Fremont, San Francisco, CA, USA). The fragments’ size was determined using Gene-Ruler™ 100 bp Ladder Plus (Cat. No. SM0323, Thermo Fisher Scientific Inc., Waltham, MA, USA) and visualized on a MiniBis photo documentation system using a transilluminator (ECX-15M Bio Imaging Systems, Bio-Imaging Systems, Inc., Jackson, MI, USA). The obtained PCR products were purified with a GeneMATRIX Short DNA Clean-Up Purification Kit (Cat. No. E3515, EURx Ltd., Gdansk, Poland) and sequenced in both directions by a PlateSeq kit (Eurofins Genomics Ebersberg, Gdansk, Germany). For all PCR products, Sanger Sequencing cycle sequencing technology (dideoxy chain termination/cycle sequencing) on an ABI 3730XL sequencing machine was used.

### 3.3. Bioinformatics and Phylogenetic Analysis

All obtained DNA sequences were manually edited and aligned by using the MUSCLE algorithm (muscle3.8.31) [27] in the MEGA v. 11.0.13 software [28]. The Basic Local Alignment Search Tool (BLAST +2.13.0 https://blast.ncbi.nlm.nih.gov/Blast.cgi, accessed on 1 May 2025) was used for *Fusarium* spp. identification among the generated sequence data. The obtained sequences from ITS1, *TEF-1α*, and *TUB* gene regions were deposited in the GenBank database https://blast.ncbi.nlm.nih.gov/Blast.cgi?PROGRAM=blastn&PAGE_TYPE=BlastSearch&LINK_LOC=blasthome (accessed on 1 May 2025) under Acc. Nos. ITS1 region-PP897820 (11 June 2024), PP897821 (11 June 2024), PP897823-PP897825 (11 June 2024), PP898068-PP898071 (23 November 2022), PP898415-PP898417 (11 June 2024), PP897819 (11 June 2024), PP897822 (11 June 2024), PP901862 (11 June 2024), PP903616 (11 June 2024), PP911639 (11 June 2024); *TEF-1α*-PQ408031-PQ408042 (28 September 2024), PQ417913-PQ417914 (1 October 2024); *β-TUB*-PQ479143-PQ479157 (17 October 2024), and IGS - PQ505497-PQ505513 (23 October 2024). The phylogenetic relationships among the Bulgarian isolates and the most similar sequences of other countries’ isolates available in GenBank were performed using the MEGA v. 11.0.13 software [28]. Although all four loci (ITS1, *TEF-1α*, *β-TUB*, and IGS) were successfully sequenced, only ITS1 and *TEF-1α* were selected for phylogenetic analysis, as they produced high-quality alignments and reliable species-level resolution. By contrast, *β-TUB* sequences often presented alignment issues, likely due to the presence of paralogous gene copies [29], while IGS sequences exhibited high variability and frequent recombination, which affected alignment stability across isolates [30].

Furthermore, the BLASTn search results for the four loci were not consistent across all isolates, and public reference sequences for *β-TUB* and IGS in GenBank were either insufficient or ambiguous. Given these limitations, a combined phylogenetic tree incorporating all four markers was not practical. For this reason, phylogenetic trees were constructed using only ITS1 and *TEF-1α*—two loci that are widely accepted for delineating *Fusarium* spp. boundaries [12,31].

While we acknowledge that a combined ITS1 + *TEF-1α* multilocus tree could further corroborate these results, our approach aimed to independently assess the discriminatory power of each marker. The separate trees generated using the same reference sequences showed strong topological congruence and high bootstrap support, validating the robustness of both loci. *TEF-1α*, in particular, provided superior resolution within the *Fusarium fujikuroi* species complex (FFSC), aligning consistently with species boundaries and toxin gene profiles.

The evolutionary history was deduced via the maximum likelihood approach employing the Tamura 3-parameter model with a discrete gamma distribution (TN93 + G) [32] in MEGA v11.0.13. To assess branch support, 1000 bootstrap replicates were conducted, and bootstrap values ≥ 50 were displayed at the corresponding internal nodes on the phylogenetic trees. All instances with gaps or missing data were removed using complete deletion. This analysis was applied to ITS1 and *TEF-1α* sequences to infer the evolutionary relationships among the detected *Fusarium* spp.

## 4. Results

### 4.1. Molecular Identification of Fusarium spp. Using Four-Locus Barcoding

Seventeen *Fusarium* isolates were recovered from maize kernels across three key agricultural regions in Bulgaria. Initial morphological screening suggested the presence of multiple *Fusarium* spp., but definitive species delineation was achieved using a multilocus DNA barcoding approach involving the ITS1, IGS, *TEF-1α*, and *β-TUB* loci. Each barcode provided complementary resolution, with *TEF-1α* and ITS1 markers yielding the most reliable identifications at the species level, consistent with previous reports [12,31].

Sequence analysis of the *TEF-1α* region—considered, per O’Donnell et al. [31], the secondary barcode for *Fusarium* spp.—allowed for the confident identification of *F. proliferatum* (*n* = 9), *F. verticillioides* (*n* = 3), *F. oxysporum* (*n* = 1), and *F. subglutinans* (*n* = 1). ITS1 analysis detected an additional species, *F. sporotrichioides*, highlighting its value for detecting species within the FSAMSC complex [12,30].

IGS and *β-TUB* regions offered further resolution, although with limitations. The TUB locus exhibited lower discriminatory power for some closely related strains due to paralogous sequences, as reported by Gálvez et al. [29]. The IGS region was prone to sequence polymorphisms and recombination, which limited its effectiveness for some taxa [30].

Overall, the multilocus strategy enabled accurate assignment of all 17 isolates to six species, including 2 isolates that showed ambiguous or equal matches to both *F. fujikuroi* and *F. proliferatum*. These were classified under the *F. fujikuroi* species complex (FFSC), a known hotspot for cryptic diversity [13].

### 4.2. Phylogenetic Resolution and Geographic Patterns

**Figure 2:**
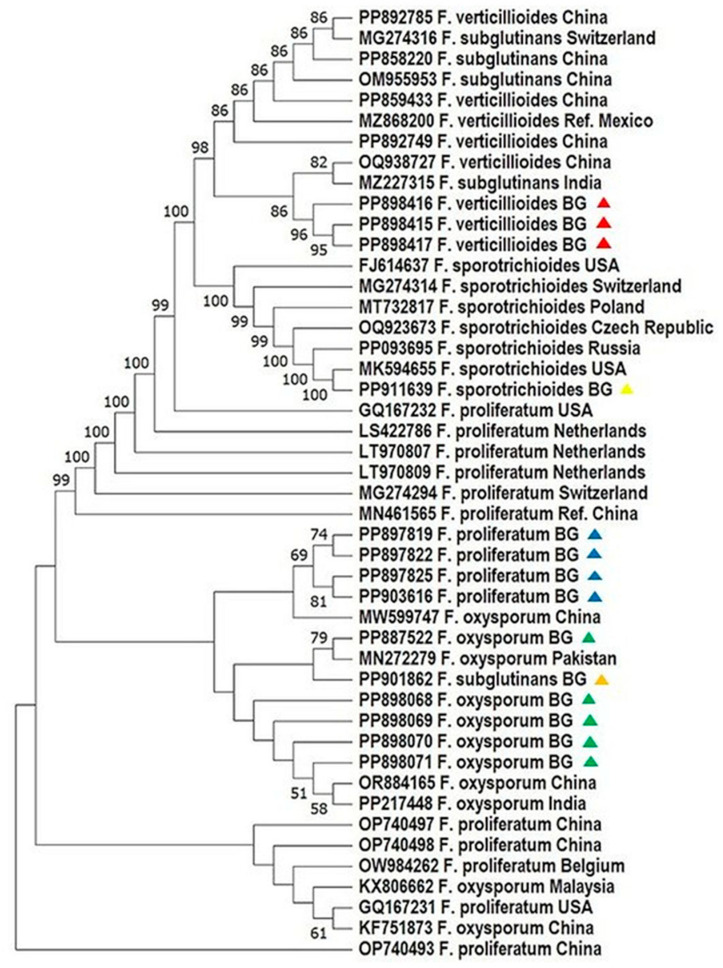
Phylogenetic analysis of *Fusarium* spp. based on the sequence analysis of the ITS region in the MEGA 11.0 software [28]. A phylogenetic tree was inferred through the maximum likelihood approach, applying the Tamura three-parameter substitution model combined with a discrete gamma distribution (TN93 + G) [32]. Support for branches was evaluated using 1000 bootstrap replicates, with bootstrap values of 50 or higher shown at the respective nodes. Positions with gaps or missing data were removed entirely (complete deletion method). Each sequence is labeled with the organism’s name followed by its GenBank accession number. The following color scheme is used: *F. verticillioides*—red; *F. proliferatum*—blue; *F. sporotrichioides*—yellow; *F. oxysporum*—green; *F. subglutinans*—orange.

**Figure 3:**
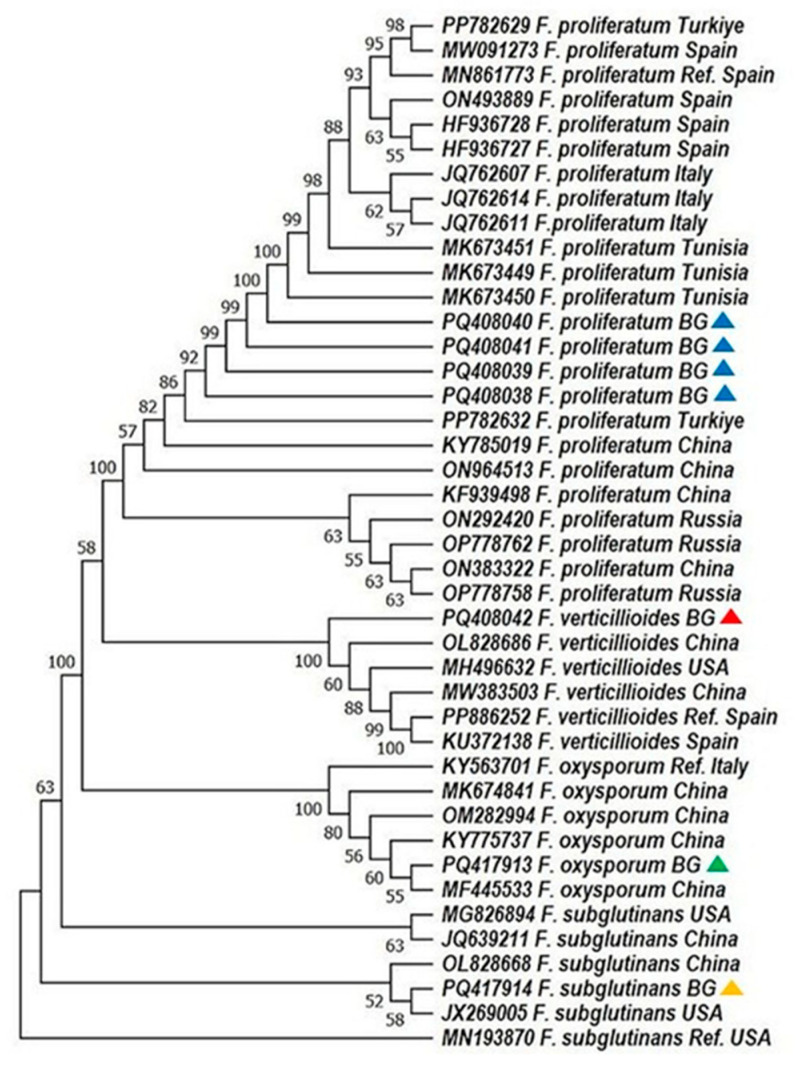
Phylogenetic analysis of *Fusarium* spp. based on the sequence analysis of the translation elongation factor (*TEF-1α gene*) in the MEGA 11.0 software [28]. A phylogenetic tree was inferred through the maximum likelihood approach, applying the Tamura three-parameter substitution model combined with a discrete gamma distribution (TN93 + G) [32]. Support for branches was evaluated using 1000 bootstrap replicates, with bootstrap values of 50 or higher shown at the respective nodes. Positions with gaps or missing data were removed entirely (complete deletion method). Each sequence is labeled with the organism’s name followed by its GenBank accession number. The following color scheme was used: *F. verticillioides*—red; *F. proliferatum*—blue; *F. oxysporum*—green; *F. subglutinans*—orange.

Phylogenetic analyses based on ITS1and *TEF-1α* sequences revealed high bootstrap support and clear species-level clustering. Bulgarian *F. proliferatum* isolates formed a tight clade, genetically close to Turkish and Tunisian strains, supporting a possible regional lineage (Figure 2 and Figure 3; [31]). In contrast, *F. verticillioides* and *F. subglutinans* isolates clustered with reference sequences from China and the USA, suggesting potential introduction via grain trade—a hypothesis echoed in similar studies on maize pathogens [33,34,35].

Notably, *F. sporotrichioides*, identified exclusively via ITS1 barcoding, grouped with European and American strains, confirming its phylogenetic distinctiveness within the dataset. These results underscore the necessity of using both protein-coding and non-coding loci for reliable species delineation, particularly in complex or globally distributed taxa [12,31,36].

### 4.3. Mycotoxigenic Gene Detection by Multiplex PCR

All 17 isolates were screened for toxigenic potential using a newly optimized multiplex PCR system. The assay successfully amplified biosynthetic gene targets associated with fumonisins (*fum6*, *fum8*), trichothecenes (*tri5*, *tri6*), and zearalenone (*zea2*) (Table S2), following the degenerate primer protocol of Dawidziuk et al. [17].

Fumonisin-associated genes were detected in 94% of the isolates, with *fum6* present in all and *fum8* absent in only one isolate (*F. proliferatum* strain 191). ZEA-related genes were amplified in 17.6% of isolates, particularly those of *F. oxysporum*, corroborating emerging evidence that this species may produce ZEA under specific conditions [37,38,39].

Only a single isolate (*F. proliferatum* strain 191) tested positive for trichothecene biosynthetic genes (*tri5*, *tri6*), which is atypical, as trichothecene production is generally associated with *F. graminearum* and *F. sporotrichioides* [40]. Mixed gene profiles were detected in 18% of isolates—most notably combinations of FUMs with ZEA or trichothecenes—raising food safety concerns due to potential co-contamination.

The multiplex system proved efficient for simultaneous detection of multiple toxin biosynthetic genes, reinforcing its potential utility in high-throughput screening of *Fusarium*-infected cereals.

## 5. Discussion

The accurate identification and risk assessment of *Fusarium* species in maize are vital for ensuring food safety and guiding management strategies against mycotoxin contamination. The implementation of multilocus barcoding—ITS1, IGS, *TEF-1α*, and *β-TUB*—proved essential for resolving species within closely related complexes, particularly the *Fusarium fujikuroi* species complex (FFSC) and *F. oxysporum* species complex (FOSC). While ITS1 and *TEF-1α* provided consistent species-level resolution in most cases, *β-TUB* and IGS added discriminatory support or helped clarify ambiguous matches, aligning with previous findings that *TEF-1α* is the most reliable single marker for *Fusarium* species identification [31].

Further supporting this conclusion, our ITS1 and *TEF-1α* phylogenies—constructed using identical reference sequences—exhibited strong congruence and consistent species clustering. Although we did not construct a combined ITS + *TEF-1α* phylogeny, the high agreement between the two single-locus trees reinforces the diagnostic value of TEF-1α as a standalone marker. Future studies may benefit from concatenated phylogenetic analyses to enhance fine-scale taxonomic resolution.

Nevertheless, due to known issues such as polymorphisms and paralogous copies in the *β-TUB* and IGS regions [29,30], these loci are best employed in combination with others rather than used independently.

This multilocus approach resolved species identities in cases where single markers yielded conflicting BLAST results. For instance, isolates 34 and 190 were classified within the FFSC based on equal identity matches to *F. proliferatum*, *F. fujikuroi*, or *F. verticillioides*, illustrating the limitations of single-locus identification in genetically overlapping taxa [13]. Such ambiguities highlight the importance of multilocus genotyping in differentiating between species with overlapping ecological niches or genetic backgrounds.

Phylogenetic analyses based on ITS1 and *TEF-1α* sequences further revealed geographic clustering of Bulgarian isolates with strains from Asia, North Africa, and Southern Europe. *F. proliferatum* isolates, in particular, exhibited close affinity to Turkish and Tunisian strains, consistent with trade-related transmission pathways. Conversely, the broader dispersion of *F. oxysporum*, *F. verticillioides*, and *F. subglutinans* across different clades suggests multiple introduction sources and ongoing population divergence—observations consistent with studies from Europe and China [32,33,34].

From a food safety perspective, the novel multiplex PCR assay offers a practical solution for rapid screening of multiple toxin biosynthesis genes. Detection of fumonisin biosynthetic genes in 94% of isolates supports the dominance of FUMs as the most common *Fusarium*-derived mycotoxins in maize, in line with previous global reports [35,36]. Interestingly, the finding that *F. oxysporum* isolates carried *zea2* (zearalenone synthase) supports emerging data identifying this species as a potential ZEA producer, although historically it was not considered a major mycotoxin contributor [37,38,39]. Similarly, the rare detection of trichothecene genes in one *F. proliferatum* isolate (strain 191) suggests sporadic horizontal gene acquisition or cryptic potential in non-traditional TRI producers, echoing findings from recent comparative genomic studies [40,41,42].

Notably, 18% of isolates exhibited the potential for eventual co-production of FUMs and ZEA or TRI toxins, underscoring the growing concern over mixed mycotoxin contamination in maize-based foods and feeds. Such co-contamination is increasingly reported and has been linked to cumulative toxic effects on animal and human health [8,43,44,45].

It is crucial to underscore that the multiplex PCR assay identifies the genetic capacity for mycotoxin biosynthesis, rather than the expression or production quantities. Although the degenerate primers used were previously validated for specificity and performance, our protocol was not compared directly with quantitative methods like LC-MS, qPCR, or ELISA. These validations will be prioritized in future studies to strengthen the assay’s practical diagnostic value.

In addition, the phylogenetic groupings observed in the ITS1- and *TEF-1α*-based trees showed strong concordance with the results of the multiplex PCR assay for mycotoxin biosynthesis gene detection. Isolates classified within the *Fusarium fujikuroi* species complex (e.g., *F. proliferatum* and *F. verticillioides*) also carried fumonisin biosynthesis genes (*fum6*, *fum8*), while those outside this clade—such as *F. sporotrichioides*—contained *tri* or *zea* genes, in line with their known mycotoxigenic profiles [6,33]. This correspondence between phylogenetic identity and biosynthetic gene presence supports the robustness of the combined molecular approach for both accurate species identification and early risk evaluation of potential toxin contamination. The combination of multilocus barcoding and gene-targeted multiplex PCR significantly improves the precision and efficiency of *Fusarium* diagnostics. This dual-system approach can serve as a model for integrated monitoring frameworks aimed at early detection, species-specific surveillance, and prevention of multi-mycotoxin risks in post-harvest grains.

## 6. Conclusions

This study introduces a molecular protocol combining multilocus DNA barcoding and a multiplex PCR system to improve the identification and toxigenic profiling of *Fusarium* spp. in post-harvest maize grains. By targeting four genetic loci—ITS1, *TEF-1α*, *β-TUB*, and IGS—the barcoding strategy enabled effective species-level classification, with ITS1 and *TEF-1α* offering the highest phylogenetic resolution. In parallel, a multiplex PCR assay was developed for simultaneous detection of mycotoxin biosynthesis genes (*fum6*, *fum8*, *tri5*, *tri6*, and *zea2*), allowing for efficient screening of toxigenic potential.

The findings revealed a predominance of *F. proliferatum* and widespread presence of fumonisin genes among isolates, with several strains also carrying genes for trichothecene and zearalenone synthesis. The dual molecular approach provides a reliable tool for early detection and risk assessment of mycotoxigenic *Fusarium* spp. contamination in maize. It offers practical application potential in food safety monitoring and post-harvest management strategies.

Future work should explore integrating this protocol into broader surveillance systems and correlating gene presence with actual mycotoxin levels under different agronomic conditions.

## Figures and Tables

**Table 1:** Primers used in the study for *Fusarium* spp. identification.

Locus	Primer Sequence (5′-3′)	PCR Products Size (bp)	Primers Annealing T (°C)	Reference
Internal transcribed spacer 1 region (ITS1)	NS1: 5′-TCCGTAGGTGAACCTGCG G-3′ NS2: 5′-TCCTCCGCTTATTGATATGC-3′	544	52.5	White et al. [24]
Translational elongation factor 1α (*TEF-1α*)	EF1: 5′-ATGGGTAAGGAAGACAAGAC-3′ EF2: 5′-GGAAGTACCAGTGATCATGTT-3′	700	54.0	O’Donnell et al. [25]
β-tubulin (*TUB*)	BTAFR: 5′-CGTCTAGAGGTACCCATACCGGCA-3′ BT5: 5′-GCTCTAGACTGCTTTCTGGCAGACC-3′	600	51.1	Tooley et al. [26]
Intergenic spacer region (IGS)	CNL12: 5′-CGCACGTATAGATGGACAAG-3′ CNS1: 5′-GGCGAAGGACGGCTTAC-3′	200	56.6	Jurado et al. [15,16]

**Table 2:** The list of primers used to detect the mycotoxin potential of the *Fusarium* spp. isolated from maize kernel to produce fumonisin B1, trichothecenes, and zearalenone.

Target Gene	Primer Name	Sequences (5′-3′)	Product Length (bp)	Annealing T (°C)
Trichodiene synthase (*tri5*)	T5_am_fA1	CTY MRR ACM ATY GTN GGC ATG	468	54.0
	T5_am_rA1	AVA CCA TCC AGT TYT CCA TYT G		
Zinc finger transcription factor (*tri6*)	TRI6_dm_fA2	TAT GAA TCA CCA ACW TTC GA	526	54.0
	TRI6_dm_rA1	CGC CTR TAR TGA TCY CKC AT		
Zearalenone polyketide synthase (*zea2*)	ZEA2_dm_fA1	ACM TCA CCA TCM AAR TTC TG	340	51.9
	ZEA2_dm_rA1	GCR TCY CKG TAR TCR CTC AT		
Oxygenase (*fum6*)	FUM6_dm_fA2	CRA CMG AGA TCA TGG TGA C	672	51.4
	FUM6_dm_rA1	GTY TCR TGT CCK GCA ATG AG		
Oxoamine synthase (*fum8*)	F8_am_fA1	GGY TCK TTT GAG TGG TGG C	800	51.4
	F8_am_rA1	CRA CWG GAA ARC AKA YRA YGG		

Note: *tri6* encodes a zinc finger transcription factor regulating trichothecene synthesis, while *tri5* encodes trichodiene synthase, the key enzyme catalyzing the first committed step in trichothecene biosynthesis.

## Data Availability

Data are available from the authors upon request.

## Supplementary Materials

The following supporting information can be downloaded at: https://www.mdpi.com/article/10.3390/mps8040078/s1, Table S1: *Fusarium* isolates with geographically localization and GenBank accession numbers used in the phylogenetic analysis; Table S2: Mycotoxigenic profile of *Fusarium* spp. isolated from maize grains in Bulgaria.
