# Supplementary material for: Novel DNA Barcoding and Multiplex PCR Strategy for the Molecular Identification and Mycotoxin Gene Detection of Fusarium spp. in Maize from Bulgaria
Source: Methods Protoc. 2025 Jul 9;8(4):78. doi: 10.3390/mps8040078 (PMC12286001; doi:10.3390/mps8040078)
Supplement: Supplementary file 1 [file mps-08-00078-s001.zip › mps-3662561-supplementary.pdf]

Supplementary

Table S1. *Fusarium* isolates with geographically localization and GenBank accession numbers used in the phylogenetic analysis.

Species	Isolate	Location	GenBank accession no.			
			ITS rDNA	*TEF-1α*	IGS	*TUB*
F. proliferatum	ITS 34	Pleven	PP897819			
F. oxysporum	ITS 35	Stara Zagora	PP898068			
F. oxysporum	ITS 36	Stara Zagora	PP898069			
F. proliferatum	ITS 37	Pleven	PP897820			
F. oxysporum	ITS 38	Shumen	PP898070			
F. proliferatum	ITS 39	Shumen	PP897821			
F. proliferatum	ITS 40	Pleven	PP897822			
F. proliferatum	ITS 41	Stara Zagora	PP897823			
F. proliferatum	ITS 42	Pleven	PP898071			
F. proliferatum	ITS 43	Shumen	PP897824			
F. verticillioides	ITS 44	Pleven	PP898415			
F. subglutinans	ITS 45	Stara Zagora	PP901862			
F. verticillioides	ITS 187	Stara Zagora	PP898416			
F. verticillioides	ITS 188	Stara Zagora	PP898417			
F. proliferatum	ITS 189	Shumen	PP903616			
F. proliferatum	ITS 190	Pleven	PP897825			
F. sporotrichioides	ITS 191	Pleven	PP911639			
F. proliferatum	TEF 79	Pleven				
F. proliferatum	TEF 80	Stara Zagora		PQ408031		
F. proliferatum	TEF 81	Stara Zagora		PQ408032		
F. proliferatum	TEF 82	Pleven		PQ408033		
F. oxysporum	TEF 83	Shumen		PQ417913		
F. proliferatum	TEF 84	Shumen		PQ408034		
F. proliferatum	TEF 85	Pleven		PQ408035		
F. proliferatum	TEF 86	Stara Zagora		PQ408036		
F. proliferatum	TEF 87	Pleven		PQ408037		

F. proliferatum	TEF 88	Shumen	PQ408038		
F. verticillioides	TEF 89	Pleven	PQ408042		
F. subglutinans	TEF 90	Stara Zagora	PQ417914		
F. proliferatum	TEF 198	Stara Zagora			
F. proliferatum	TEF 199	Stara Zagora	PQ408039		
F. proliferatum	TEF 200	Shumen	PQ408040		
F. proliferatum/ F. fujikuroi	TEF 201	Pleven			
F. proliferatum	TEF 202	Pleven	PQ408041		
F. fujikuroi	IGS169	Pleven		PQ505511	
F. proliferatum	IGS170	Stara Zagora		PQ505497	
F. proliferatum	IGS171	Stara Zagora		PQ505498	
F. fujikuroi	IGS172	Pleven		PQ505512	
F. oxysporum	IGS173	Shumen		PQ505506	
F. proliferatum	IGS174	Shumen		PQ505499	
F. proliferatum	IGS175	Pleven		PQ505500	
F. proliferatum	IGS176	Stara Zagora		PQ505501	
F. proliferatum	IGS177	Pleven		PQ505502	
F. proliferatum	IGS178	Shumen		PQ505503	
F. verticillioides	IGS179	Pleven		PQ505508	
F. subglutinans	IGS180	Stara Zagora		PQ505507	
F. verticillioides	IGS220	Stara Zagora		PQ505509	
F. verticillioides	IGS221	Stara Zagora		PQ505510	
F. proliferatum	IGS222	Shumen		PQ505504	
F. fujikuroi	IGS223	Pleven		PQ505513	
F. proliferatum	IGS224	Pleven		PQ505505	
F. fujikuroi	TUB124	Pleven			
F. proliferatum	TUB125	Stara Zagora			PQ479143
F. proliferatum	TUB126	Stara Zagora			PQ479144
F. fujikuroi	TUB127	Pleven			PQ479156
F. proliferatum	TUB128	Shumen			PQ479145

F. proliferatum	TUB129	Shumen	PQ479146
F. proliferatum	TUB130	Pleven	PQ479147
F. proliferatum	TUB131	Stara Zagora	PQ479148
F. proliferatum	TUB132	Pleven	PQ479149
F. proliferatum	TUB133	Shumen	PQ479150
F. verticillioides	TUB134	Pleven	PQ479154
F. subglutinans	TUB135	Stara Zagora	
F. verticillioides	TUB209	Stara Zagora	PQ479155
F. proliferatum	TUB210	Stara Zagora	PQ479151
F. proliferatum	TUB211	Shumen	PQ479152
F. fujikuroi	TUB212	Pleven	PQ479157
F. proliferatum	TUB213	Pleven	PQ479153

Abbreviations: ITS1 - internal transcribed spacer 1 region; *TEF-1α* - translational elongation factor 1α; *TUB* - β-tubulin; IGS - intergenic spacer region

Supplementary Table S2. Mycotoxigenic profile of *Fusarium* spp. isolated from maize grains in Bulgaria.

Sample number	*Fusarium* species	Collection	Year of isolation	Fumonisin B1 (*fum6*)	Fumonisin B1 (*fum8*)	Trichothecene (*tri5*)	Zearalenone (*zea2*)	ITS1	*TEF-1α*	*TUB*	IGS
1.	*F. fujiκuroi*	34	2022	+	+				+	+	+
2.	*F. proliferatum*	35	2022	+	+				+	+	+
3.	*F. proliferatum*	36	2022	+	+				+	+	+
4.	*F. fujiκuroi*	37	2022	+	+				+	+	+
5.	*F. oxysporum*	38	2022	+	+			+	+		+
6.	*F. proliferatum*	39	2022	+	+			+	+	+	+
7.	*F. proliferatum*	40	2022	+	+			+	+	+	+
8.	*F. proliferatum*	41	2022	+	+			+	+	+	+
9.	*F. oxysporum*	42	2022	+	+		+	+	+	+	+
10.	*F. proliferatum*	43	2022	+	+			+	+	+	+

11.	*F. verticillioides*	44	2022	+	+		+	+	+	+
12.	*F. subglutinans*	45	2022	+	+			+	+	+
13.	*F. verticillioides*	187	2022	+	+		+		+	+
14.	*F. verticillioides*	188	2022	+	+		+			+
15.	*F. oxysporum*	189	2022	+	+		+	+	+	+
16.	*F. fujiкuroi*	190	2022	+	+			+	+	+
17.	*F. proliferatum*	191	2022	+		+		+	+	+

Abbreviations: ITS1 - internal transcribed spacer 1 region**;** ***TEF**-1α* - translational elongation factor 1α; ***TUB* -** *β-tubulin***; IGS -** intergenic spacer region
